# Global Trends in Extracorporeal Membrane Oxygenation Support for Circulatory Failure: A Bibliometric Analysis

**DOI:** 10.3390/healthcare13121365

**Published:** 2025-06-06

**Authors:** Hanming Gao, Kaihuan Zhou, Yin Chen, Yicong Ling, Qianqian Qin, Junyu Lu

**Affiliations:** 1 Department of Critical Care Medicine, The Second Affiliated Hospital of Guangxi Medical University, Nanning 530000, China; hanminggao@sr.gxmu.edu.cn (H.G.); khzhou@sr.gxmu.edu.cn (K.Z.); lyc45491314@sr.gxmu.edu.cn (Y.L.);; 2 Department of Critical Care Medicine, Cenxi People’s Hospital, Cenxi 543200, China

**Keywords:** extracorporeal membrane oxygenation, circulatory failure, bibliometrics, cardiogenic shock, mechanical circulatory support, extracorporeal cardiopulmonary resuscitation

## Abstract

Objectives: This study utilized bibliometric and visualization analyses to explore global research trends and identify research hotspots in extracorporeal membrane oxygenation (ECMO) for circulatory support to provide references and guidance for future research. Methods: This study was based on data from the Web of Science Core Collection, covering the period from 1945 to 1 August 2024. Bibliometric tools, such as VOSviewer and CiteSpace, were used to visualize the analysis of countries/regions, institutions, journals, co-cited references, and keywords in the relevant literature. Results: A total of 14,804 valid papers were included in the study. The research interest in ECMO support for circulatory failure has increased annually, with the United States being the most active in this field. The U.S. occupies most of the top journals and institutions, leading in both the volume of publications and the intensity of international collaboration. Although China has a relatively high number of publications, it lags significantly in international collaboration and representation in top journals. Keyword and citation burst analysis indicates that research on cardiac arrest, post-cardiac surgery circulatory failure, left ventricular unloading, and prognostic factors have been the focus of recent studies and are prevalent in highly impactful literature. Conclusion: The research interest in ECMO support for circulatory failure continues to rise, particularly in cardiac arrest, post-cardiac surgery circulatory failure, left ventricular unloading, and prognostic factors. Future research should investigate these key areas and optimize techniques to enhance the clinical outcomes.

## 1. Introduction

Extracorporeal membrane oxygenation (ECMO) is an advanced extracorporeal circulation technology that facilitates oxygen exchange and carbon dioxide removal by directing a portion of the patient’s blood into an external artificial heart-lung system [1]. ECMO plays a critical role in the treatment of circulatory failure. Since its introduction in 1970, the application scope of ECMO has continuously expanded, while its predecessor, cardiopulmonary bypass (CPB), had been used in cardiac surgery as early as 1954, indicating that ECMO was designed to support circulation from its inception [2]. With the accumulation of clinical experience and technological advancements, the application of ECMO in circulatory failure has broadened to include various situations such as cardiogenic shock, septic shock, postoperative low cardiac output, cardiac arrest, and heart transplantation [3,4,5]. Currently, compared with its use in respiratory failure, ECMO is widely applied in the treatment of circulatory failure. According to data from the Extracorporeal Life Support Organization (ELSO), of the 229,288 registered cases as of August 16, 2024, 119,543 cases (53.14%) were treated for circulatory failure [6].

Despite the significant clinical value of ECMO in circulatory support therapy, its widespread application faces numerous challenges and controversies. First, the high incidence of complications during ECMO application significantly increases the treatment complexity and patient mortality risk including issues such as bleeding, thrombosis, limb ischemia, and infection [4,7,8]. Second, patient selection and prognostic evaluation remain challenging due to a lack of unified standards and high-quality randomized controlled trials, making it difficult for clinicians to determine which patients are most likely to benefit from ECMO in practice [9,10,11]. Additionally, the choice of different ECMO configurations (e.g., central cannulation versus peripheral cannulation) and the complexity of anticoagulation management have sparked extensive debate and controversy in clinical practice [12,13,14]. Despite the continuous emergence of new technologies and improved treatment strategies, ECMO’s impact on long-term survival rates remains limited [11,15]. Therefore, a deep understanding of the current status and challenges of ECMO in circulatory support therapy is crucial for optimizing its clinical application.

Bibliometrics and visualization analysis, as tools of quantitative research methodology, are well-suited to reveal the knowledge structure, evolution of research topics, and future development potential in specific medical domains [16]. The primary objective of this study is to conduct a focused bibliometric analysis of ECMO used for circulatory failure, which remains underrepresented in the previous literature. While earlier bibliometric studies have often examined ECMO in general or prioritized its respiratory support role (e.g., veno-venous extracorporeal membrane oxygenation (VV-ECMO)), few have specifically explored the circulatory support domain. Additionally, limited attention has been given to thematic evolution, regional output, and international collaboration patterns. By addressing these gaps, our study seeks to identify key contributors, research hotspots, collaboration structures, and emerging trends in ECMO for circulatory failure. This is particularly important in the post-COVID-19 era, which has seen a surge in global demand for mechanical circulatory support. The findings from this study are expected to provide clinicians and researchers with valuable data-driven insights into the development of ECMO therapy, inform future clinical guidelines, and guide research directions in this critical area.

## 2. Methods

### 2.1. Data Sources

**Figure 1:**
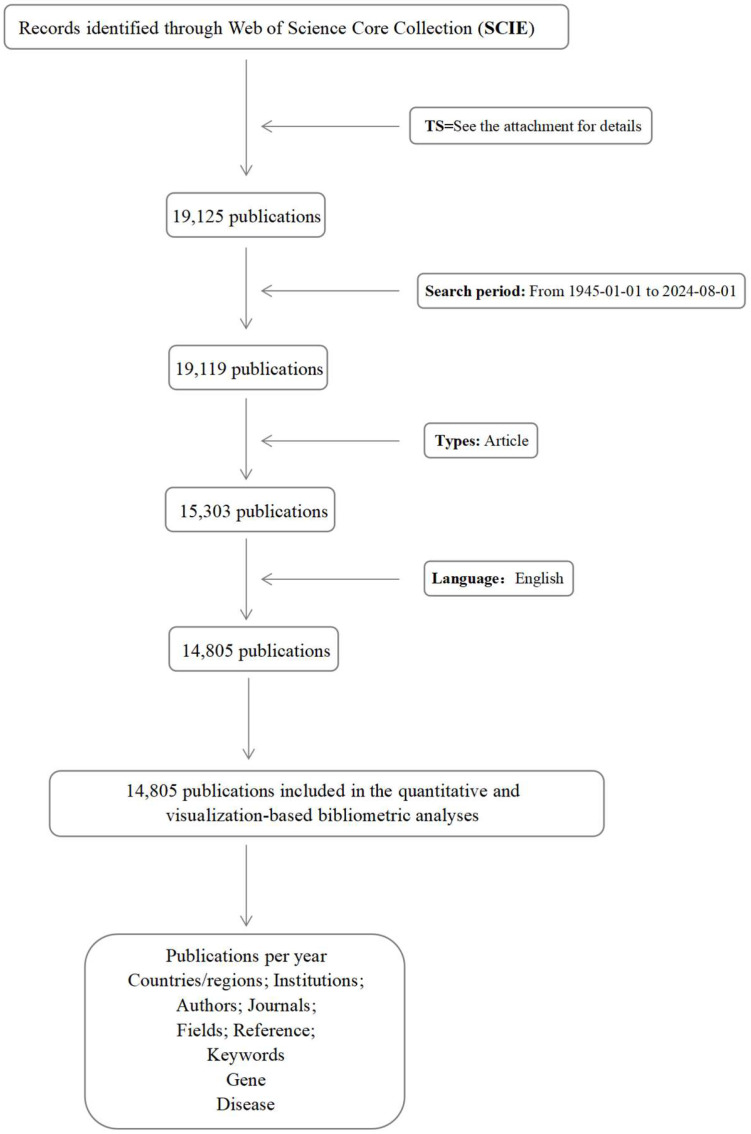
Search flowchart.

**Figure 2:**
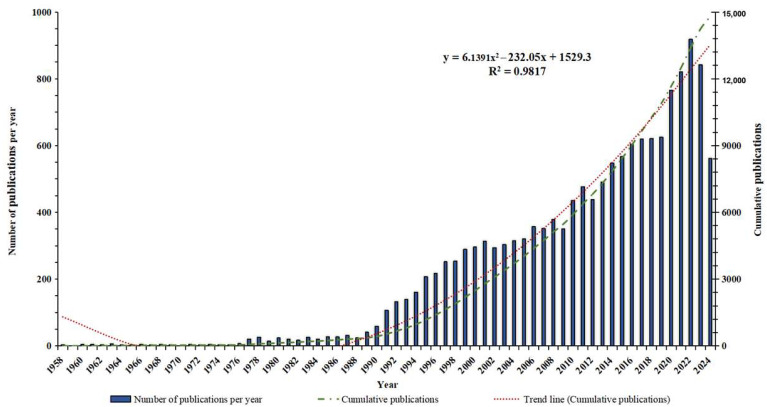
Trend analysis of global publications on ECMO support for circulatory failure.

This study used the Science Citation Index Expanded (SCIE) within the Web of Science Core Collection as the data source. Publications from 1 January 1945, to 1 August 2024, were retrieved using a Boolean logic search combining ECMO-related and circulatory failure-related terms. The search was limited to English-language publications, and the document type was restricted to “articles”. Letters, notes, book reviews, conference abstracts, and reviews were excluded. The search was completed on 8 August 2024. The search flowchart is presented in Figure 1. Although the search range began in 1945, the first eligible publication on ECMO support for circulatory failure appeared in 1958, as shown in Figure 2.

To ensure thematic relevance, two reviewers independently screened all retrieved records based on the titles and abstracts. Studies that focused exclusively on respiratory ECMO (e.g., VV-ECMO), animal experiments, or were otherwise unrelated to circulatory failure were excluded. When necessary, full-text evaluation was conducted to confirm eligibility. Ultimately, a total of 14,804 valid articles were included for analysis.

### 2.2. Data Analysis

Using bibliometric methods, we visualized the retrieved literature using VOSviewer 1.6.18 (Center for Science and Technology Studies, Leiden University, Leiden, The Netherlands), CiteSpace 6.3.R1 (Chaomei Chen, China), Pajek 64 5.16 (University of Ljubljana, Ljubljana, Slovenia), Scimago Graphica 1.0.35 (https://www.graphica.app/, accessed on 8 August 2024, USA), and Microsoft Excel (Microsoft Office 2021, Microsoft, Redmond, WA, USA). These tools were used to analyze the research status, hotspots, and trends in ECMO support for circulatory failure across countries/regions, institutions, journals, co-cited references, and keywords.

## 3. Results

### 3.1. Global Publication Trend Analysis

As presented in Figure 2, from 1 January 1958 to 1 August 2024, the cumulative number of publications on ECMO support for circulatory failure reached 14,804. From 1958 to 1990, the annual number of publications was relatively low. Between 1990 and 2001, the annual number of publications increased steadily. From 2001 to 2024, the growth in annual publications showed periodic fluctuations. The number of publications peaked in 2022, reaching 918 papers. According to the polynomial function, y = 6.1391x^2^ − 232.05x + 1529.3 (R^2^ = 0.9817, where x represents the year and y is the cumulative number of publications), created for the cumulative publication growth trend, this indicated a good fit.

### 3.2. Analysis of Publications by Country, Institution, and Journal

**Figure 3:**
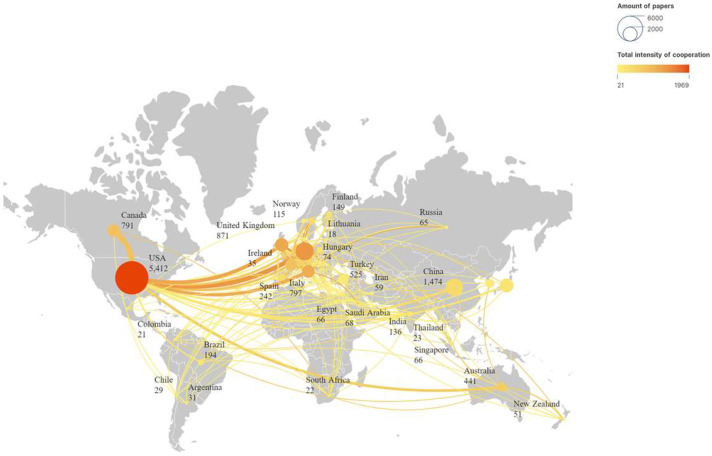
Publication and collaboration analysis of countries in ECMO support for circulatory failure research. Each circle represents a country, with the size indicating the total number of publications. The color gradient reflects the intensity of international collaboration, where darker colors denote stronger cooperation. The width and brightness of the connecting lines represent the strength of collaboration between countries. Countries with larger and darker nodes, such as the United States, show both high research output and extensive international cooperation.

We set a minimum publication threshold of 15 papers per country to analyze the publication and collaboration patterns of countries in terms of ECMO support for circulatory failure research. As shown in Figure 3, the USA had the highest total link strength value at 1969. This indicates that the USA has the strongest willingness to collaborate with other countries/regions, with the closest collaboration being with Canada. The USA also leads in the number of publications, totaling 5412 papers, followed by Germany with 1601 papers. China ranked third with 1474 papers, a high publication count, and a relatively poor collaboration willingness, with a total link strength of only 417 and ranked 12th in collaboration intensity.

**Figure 4:**
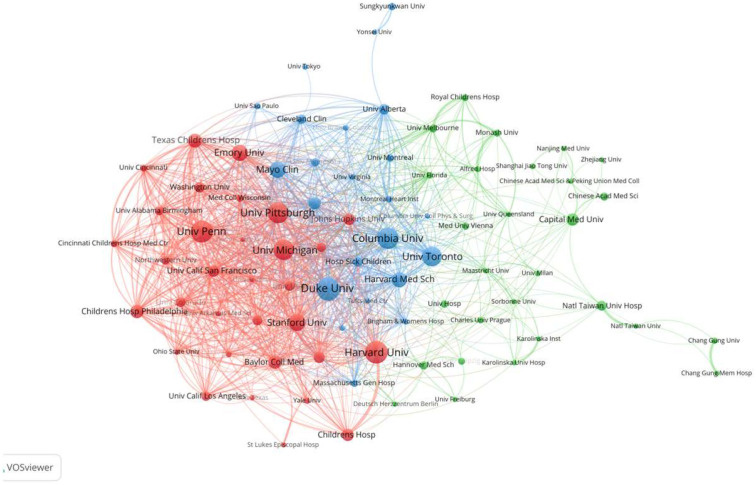
Publication and collaboration analysis of institutions in ECMO support for circulatory failure research. Each node represents a research institution, with the node size proportional to its publication count. Lines between nodes represent co-authorship links, with thicker lines indicating stronger collaboration. Colors represent clusters of institutions that collaborate more frequently, identified through co-authorship network analysis using VOSviewer. Clustering was performed using the modularity-based clustering algorithm embedded in the software.

In addition, we included 8559 institutions and set a minimum publication threshold of 55 papers. Through VOSviewer software 1.6.18 analysis, we obtained the publication and collaboration analyses of institutions. As shown in Figure 4, the University of Michigan in the red cluster had the highest total link strength. Among all institutional collaborations, the University of Pennsylvania and the Children’s Hospital of Philadelphia had the highest collaboration strength. Duke University, within the blue cluster, had the highest publication count, with 255 papers. In the green cluster, Capital Medical University had the highest publication count, with 134 papers and a total link strength of 41. Led by Capital Medical University, institutions such as the Chinese Academy of Medical Sciences and Sun Yat-sen University have formed an academic exchange network.

**Figure 5:**
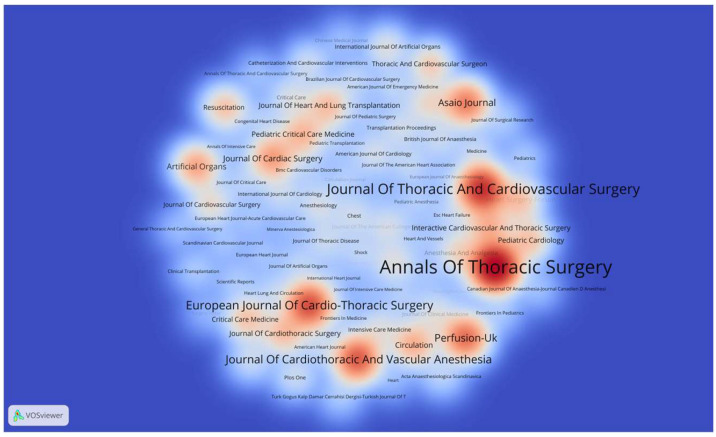
Heatmap of journal publications on ECMO support for circulatory failure. Each label represents a journal, with the font size proportional to the number of articles published on ECMO for circulatory failure. The color gradient reflects the publication density—red indicates journals with high publication output, while blue indicates lower frequency. Journals such as *Annals of Thoracic Surgery* and *Journal of Thoracic and Cardiovascular Surgery* were among the most productive in this field.

Moreover, a visual analysis of journal publications was conducted using VOSviewer software 1.6.18, including 1172 journals, with a minimum publication threshold of 30 papers, resulting in the journal publication heatmap. As shown in Figure 5, the journal with the highest number of publications was the Annals of Thoracic Surgery, with 1030 papers. The Journal of Thoracic and Cardiovascular Surgery ranked second, with 725 papers. The European Journal of Cardio-Thoracic Surgery ranked third, with 568 papers. Among the top 100 journals by publication count, approximately 42% were from the United States. China only had one journal in the top 100, the Chinese Medical Journal, which ranked 78th.

**Figure 6:**
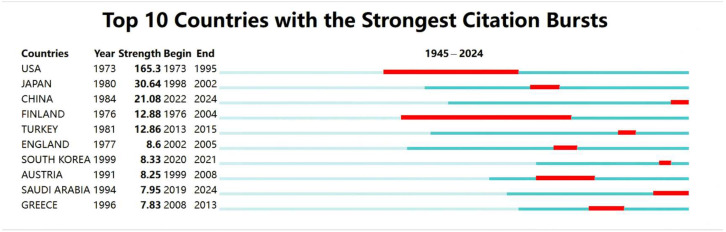
Top 10 citation bursts by country in ECMO support for circulatory failure research. Each bar represents a country’s citation activity timeline. The red segments indicate time periods during which that country’s ECMO-related publications experienced a significant surge in citations, known as “citation bursts”. The burst strength reflects the intensity of attention, with the United States showing the strongest burst over the longest duration.

Figure 6 presents the top 10 countries with the strongest citation bursts related to ECMO support for circulatory failure from 1 January 1945 to 1 August 2024. The red areas indicate the period when citations surged for each country. The USA experienced a significant citation surge from 1973 to 1995, with the highest burst strength of 165.3. Finland had the longest citation burst duration, lasting from 1976 to 2004, a total of 29 years. The citation bursts for most of the top 10 countries occurred before 2019, with only China, South Korea, and Saudi Arabia showing citation bursts during 2020–2024.

### 3.3. Citation Burst Analysis

**Figure 7:**
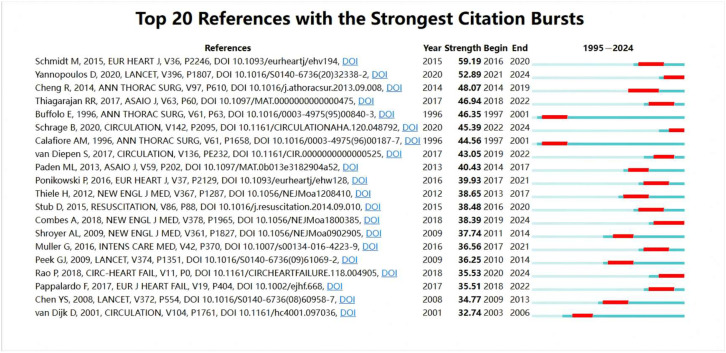
Citation burst analysis of ECMO support for the circulatory failure literature. Each row represents a reference with high citation burst strength. The red segments indicate time periods when the corresponding article received a sudden surge in citations. Citation burst strength reflects the magnitude of scholarly attention. Most of the top-bursting papers were published after 2015, indicating a recent shift in research focus. Notable references include Schmidt M et al. (2015) [17], which had the strongest citation burst strength (59.19) from 2016 to 2020, as well as Muller G et al. (2016) [18], Schrage B et al. (2020) [19], Rao P et al. (2018) [4], Combes A et al. (2018) [15], and Yannopoulos D et al. (2020) [20].

Figure 7 shows the top 20 citation burst analyses of the literature from 1 January 1995 to 1 August 2024. The red areas indicate the periods during which each paper experienced a surge in citations. Among the Top 20 cited papers, the most experienced citation bursts were between 2015 and 2024. The paper by Schmidt M et al. (2015) [17] had the highest citation burst strength, reaching 59.19, with the burst period from 2016 to 2020. This, along with Muller G et al. (2016) [18], was related to predictive models. In recent years, papers with high citation burst strengths include Schrage B et al. (2020) [19], Rao P et al. (2018) [4], Combes A et al. (2018) [15], and Yannopoulos D et al. (2020) [20].

### 3.4. Hot Keyword Analysis

**Figure 8:**
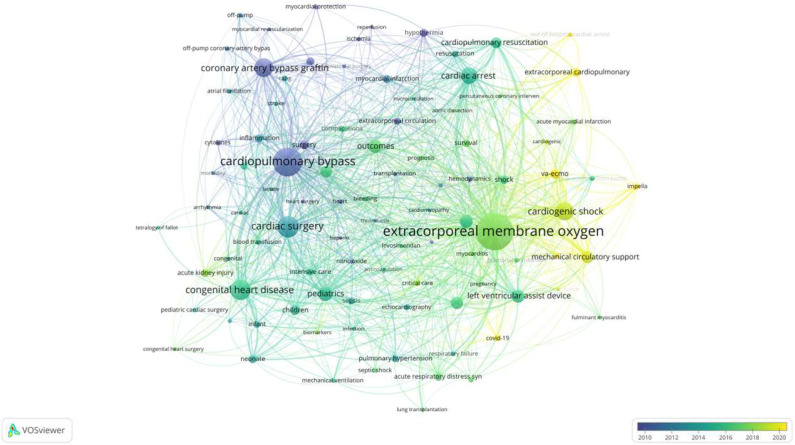
Timeline of keyword co-occurrence in ECMO support for circulatory failure. Each node represents a keyword, with size proportional to its frequency. The color gradient reflects the average publication year in which the keyword appeared: blue denotes earlier years, while yellow indicates more recent topics. The spatial distance between nodes indicates the relatedness of research themes based on co-occurrence. Clusters were identified using the modularity-based clustering algorithm integrated in VOSviewer.

Using VOSviewer software 1.6.18, a co-occurrence cluster analysis of keywords from articles published in the past 10 years was conducted. From the remaining 8642 keywords after deduplication and merging, 90 keywords were selected, with a minimum occurrence threshold of 30 times, forming a visualization map. As shown in Figure 8, keywords such as “CPB”, “cardiac arrest”, and “heart failure” appeared mainly before 2015, reflecting ECMO’s early use in surgical support and critical care rescue. Between 2015 and 2020, the rise of “cardiogenic shock” and “mechanical circulatory support” marked a shift toward broader clinical indications, especially for acute shock management. After 2020, terms like “ECPR”, “Impella”, and “out-of-hospital cardiac arrest” became more prominent, indicating growing interest in early intervention, hybrid support strategies, and prehospital ECMO deployment. This temporal trend highlights the transition from basic feasibility to advanced, patient-centered applications in circulatory failure care.

**Figure 9:**
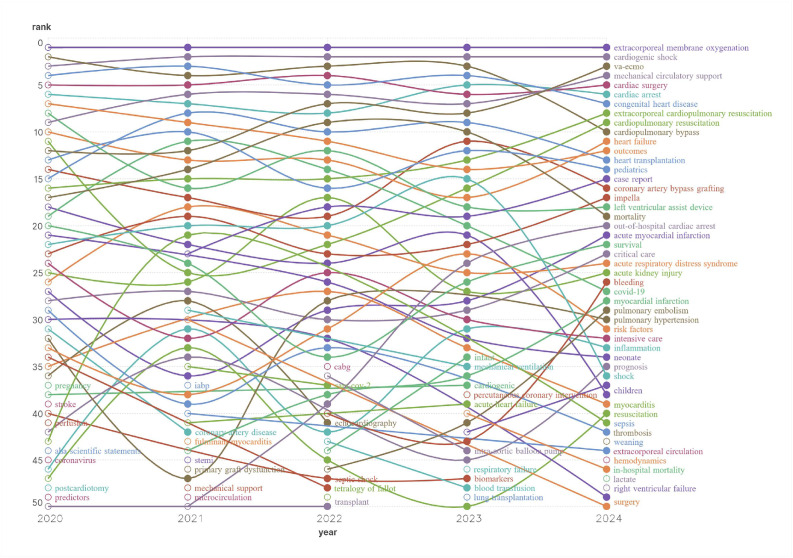
Heatmap of the top 50 keywords in ECMO support for circulatory failure over the past 5 years. The y-axis represents the keyword rank by annual frequency, and the x-axis represents the year (2020–2024). Each line tracks the change in ranking of a specific keyword over time. Keywords such as “extracorporeal membrane oxygenation”, “cardiogenic shock”, and “mechanical circulatory support” consistently remained at the top, while others showed notable fluctuations, indicating emerging or declining research trends.

Additionally, the keywords of articles from 2020 to 2024 were selected, and the top 50 keywords by frequency for each year were used to create the heatmap of keyword trends over the past 5 years. As shown in Figure 9, the top ten keywords over the past 5 years (excluding ECMO and veno-arterial extracorporeal membrane oxygenation, VA-ECMO) were as follows: cardiogenic shock, mechanical circulatory support, cardiac surgery, cardiac arrest, congenital heart disease, ECPR, cardiopulmonary resuscitation, CPB, heart failure, and outcomes. Keywords such as cardiogenic shock, cardiac arrest, cardiac surgery, and outcomes consistently maintained a high popularity. The research interest in keywords like out-of-hospital cardiac arrest and ECPR has also grown significantly.

To support the interpretation of Figure 9, Table 1 presents the quantitative frequency trends of the top 20 keywords over the past five years. Table 1 provides quantitative insights into the temporal evolution of high-frequency terms. The consistently high ranking of keywords such as “cardiogenic shock”, “cardiac arrest”, and “mechanical circulatory support” highlights their central role in the pathophysiology and management of circulatory failure. “Cardiac arrest” and “ECPR” reflect the growing attention to the extracorporeal resuscitation and emergency deployment of VA-ECMO, particularly in post-cardiac arrest scenarios. The emergence of “Impella” indicates increased exploration of mechanical unloading strategies in ECMO-supported patients. Keywords like “outcomes” and “ECMO weaning” signal a shift toward individualized care, prognostic modeling, and long-term management. Overall, the keyword trends demonstrate a transition from generalized support concepts to more refined, patient-centered, and outcome-driven ECMO research.

## 4. Discussion

### 4.1. General Information

The global publication trend on ECMO support for circulatory failure reflects a continuous rise in research interest and the expanding application of the technology in this field. However, the characteristics of publication volume in different periods vary, showcasing the different stages of ECMO technology development for treating circulatory failure. Initially, before 1990, the number of publications was relatively sparse, indicating that ECMO technology was still in its infancy. At that time, ECMO equipment was relatively primitive, the technology was immature, operations were complex, and there was a high risk of complications, leading to suboptimal treatment outcomes [21,22]. Consequently, the clinical application of ECMO was limited for a significant period. From 1990 to the early 21st century, as technology gradually improved and clinical practice expanded, the number of publications increased rapidly. In 1989, Bartlett and Toomasian established the ELSO at the University of Michigan [23]. This organization promoted the standardization and regulation of ECMO by creating registries, establishing guidelines, and providing educational training, thereby entering a new phase of rapid development. Entering the 21st century, publication volumes continued to show a fluctuating upward trend. The 2009 H1N1 influenza pandemic and the 2019 global outbreak of the COVID-19 pandemic, with infected patients often suffering from myocardial injury and cardiogenic shock [24,25,26], led to a significant number of critical cases requiring circulatory support. This notably drove the continuous rise in research interest in ECMO for treating circulatory failure. With the widespread application of ECMO technology in critical care medicine, it is expected that the research interest and academic output on ECMO support for circulatory failure will continue to increase, further advancing this field.

The United States, the birthplace of ECMO, has achieved several world firsts in this field: the first clinical application of ECMO, the establishment of the first ECMO center [27], and the formation of the first ECMO organization [23]. Analyzing publication volumes by country, journal, and institution clearly shows that the United States is undoubtedly in a leading position in the field of ECMO support for circulatory failure research. The U.S. ranked first globally with 5412 publications and demonstrates a strong willingness for international collaboration, with a total link strength of 1969. Citation burst analysis for countries revealed that the U.S. had a citation burst strength of 165.3 from 1973 to 1995. Additionally, among the top 100 journals by publication count, approximately 42% are U.S. journals, further solidifying its academic influence in this field.

China ranked third globally in publication volume in the field of ECMO support for circulatory failure, indicating high research activity and output. However, compared with other countries, China still has room for improvement in terms of international collaboration and research impact. Chinese research institutions, such as Capital Medical University, had notable domestic influence; however, it exhibited a lower collaboration intensity internationally. Despite the large publication volume, only the *Chinese Medical Journal* is ranked among the top 100 journals, in 78th place. To further enhance China’s international standing in this field, we recommend strengthening international collaboration, improving the research quality, and focusing on global research hotspots. These efforts will help to increase China’s global influence and academic standing in the field of ECMO support for circulatory failure research.

Notably, among the top 10 countries with citation bursts, the citation activity of most nations had stabilized before 2019. In contrast, China, South Korea, and Saudi Arabia experienced significant citation bursts between 2020 and 2024. This shift reflects the accelerated development of ECMO-related research in response to global health emergencies such as the COVID-19 pandemic as well as increasing demands for advanced life support technologies. While China and the United States remain the dominant contributors, other countries have also emerged as important players in this field. Germany, for instance, consistently ranked among the top five in publication volume and showed high centrality in global co-authorship networks, reflecting its sustained academic influence and active involvement in collaborative research—particularly on cardiogenic shock and mechanical circulatory support. South Korea, in addition to its recent citation burst, demonstrated rapid growth in publications focused on perioperative ECMO strategies and cardiac surgery. Saudi Arabia, though a more recent entrant, showed a steady rise in publication output and international collaboration since 2020, likely driven by increased investment in critical care infrastructure and participation in multinational research.

Collectively, these developments suggest that the global research landscape on ECMO support for circulatory failure is becoming increasingly diversified, with a broader set of countries contributing meaningfully to academic progress and international cooperation.

### 4.2. Analysis of the High-Impact Literature and Hot Keywords

Exploring the high-impact literature and hot keywords in ECMO support for circulatory failure revealed the development trajectory and key research themes in this field. Regarding the high-impact literature and hot keywords, this study noted several relevant themes in the ECMO support for circulatory failure field and their developmental progress.

#### 4.2.1. Circulatory Failure After Cardiac Arrest

Interestingly, this study found that research focused on ECMO treatment for circulatory failure following cardiac arrest occupied a significant position among the top 20 citation bursts and was particularly prominent among the hot keywords in the past five years. Globally, the incidence of cardiac arrest is approximately 30 to 97 cases per 100,000 people [28]. The overall survival rate for patients with cardiac rest is low: out-of-hospital cardiac arrest survival rates are typically below 10%, while for in-hospital cardiac arrest, the survival rate is relatively higher, approximately 23% [29]. These statistics indicate that cardiac arrest is a widespread public health crisis that requires urgent attention. As of 19 August 2022, the ELSO registered 18,660 adult ECPR cases, with only 31% of patients successfully surviving to discharge or being transferred to other institutions for care [6].

Recent studies have shown that ECPR can improve the survival rates and neurological outcomes in patients with cardiac arrest [30,31]. A 2008 observational study published in *The Lancet* indicated that for patients who experienced in-hospital cardiac arrest due to cardiac causes and had CPR lasting more than 10 min, those who received ECPR had significantly higher discharge survival rates (28.8% vs. 12.3%, *p* < 0.0001) and 1-year survival rates (18.6% vs. 9.7%, *p* = 0.006) compared with those receiving conventional CPR, confirming the efficacy of ECPR in such patients [32]. The 2015 CHEER trial combined ECMO with mechanical CPR, hypothermia therapy, and early reperfusion to explore the impact of a multimodal rescue strategy on patients with refractory circulatory failure after cardiac arrest [33]. In this trial, 26 patients participated, with 96% (25 patients) achieving return of spontaneous circulation and 54% (14 patients) having full neurological recovery (CPC score of 1) at hospital discharge. These results suggest that combining multiple therapeutic approaches can significantly improve the short-term outcomes in patients with refractory circulatory failure after cardiac arrest, marking a shift from single treatment to comprehensive rescue strategies [33]. By 2018, research focused on the long-term prognosis of ECMO in patients with cardiac arrest, with particular attention to the long-term survival rates and complications management [4]. Studies have shown that although the survival rate of patients treated with VA-ECMO is about 40% at hospital discharge, complications such as left ventricular dilation, pulmonary edema, and thrombosis still require effective management [4]. These in-depth analyses and data reflect the increasing maturity of ECMO technology and indicate that standardization in this field is gradually improving. The 2020 ARREST trial extended research to out-of-hospital cardiac arrest, validating the effectiveness of ECMO in supporting circulatory failure under extreme conditions and demonstrating its expanded application range [20]. This technique was subsequently included in the 2023 editions of the American guidelines on ECPR for cardiac arrest [34] and the Chinese Expert Consensus on Adult ECPR [35]. As mentioned in the scientific statement by the American Heart Association and the Neurocritical Care Society, although ECPR shows potential benefits for patients with cardiac arrest, the lack of sufficient clinical research evidence prevents ECPR from providing clear guideline recommendations on key treatment decisions such as oxygenation, ventilation, and blood pressure control [35]. The statement emphasizes the need for future research, particularly in areas such as patient selection for ECPR, cardiac and neurological function monitoring, pharmacotherapy, fluid management, infection control, nutritional and metabolic support, and neurological prognostication, aiming to improve the treatment outcomes and quality of life for patients with cardiac arrest [36].

#### 4.2.2. Left Ventricular Unloading

This study noted that a research paper on left ventricular unloading in ECMO treatment for cardiogenic shock showed a high citation burst intensity in the past two years, indicating it as one of the recent research hotspots [19]. Peripheral ECMO increases the pressure at the aortic root through its retrograde flow mechanism during circulatory failure treatment, leading to a significant increase in left ventricular afterload, ventricular dilation, impairment of the heart’s pumping ability, and potential structural damage to the heart [37]. Additionally, elevated left ventricular pressure can trigger arrhythmias, increase the risk of cardiac events, and potentially cause pulmonary edema, which affects the patient’s oxygenation status [38]. An international multicenter cohort study by Schrage B et al. demonstrated that left ventricular unloading significantly reduced the 30-day mortality rate in patients treated with VA-ECMO for cardiogenic shock (HR: 0.79, 95% CI: 0.63–0.98, *p* = 0.03) [19]. Although left ventricular unloading increases the risk of complications, such as severe bleeding and renal failure, the study supports it as an effective strategy for improving survival rates [19]. In further exploring the relationship between left ventricular unloading and ECMO treatment for cardiogenic shock, other studies have provided additional evidence. The EARLY-UNLOAD trial by Kim et al. found no significant difference in 30-day mortality between early left ventricular unloading and conventional strategies in patients with cardiogenic shock treated with VA-ECMO (46.6% vs. 44.8%, *p* = 0.942) [39]. However, the median time to relief from pulmonary congestion was shorter in the early unloading group compared with the conventional group (3 days vs. 5 days, *p* = 0.027). This highlights the potential importance of optimizing unloading strategies. Additionally, Baldetti et al. emphasized the critical role of the etiology of cardiogenic shock in guiding left ventricular unloading decisions, particularly for patients with cardiogenic shock related to acute myocardial infarction, where unloading may help reduce myocardial workload and promote recovery [40]. Collectively, these studies indicate that while left ventricular unloading involves certain risks, carefully selecting unloading strategies and timing can significantly improve the prognosis of patients with cardiogenic shock, providing a basis for further research and the development of personalized treatment plans.

#### 4.2.3. Cardiac Surgery-Related Circulatory Failure

The emergence of CPB technology in the mid-20th century marked a revolutionary breakthrough in the field of cardiac surgery. This technology made it possible to successfully perform complex surgeries with an arrested heart, significantly advancing the field [41]. Mechanical circulatory support technology has become an indispensable part of cardiac surgery. Among these, ECMO, as a mechanical circulatory support technology capable of providing both heart and lung support, plays a crucial role in managing circulatory failure following cardiac surgery. However, despite its increasing application, several papers related to the pediatric extracorporeal life support (PELS-1) multicenter cohort study have revealed that this technology still faces many challenges in practical clinical application [42,43,44].

First, the 2023 PELS-1 study revealed significant heterogeneity in clinical practices of extracorporeal life support (ECLS) following cardiac surgery, with differences across centers in anticoagulation management, left ventricular unloading, and ECLS monitoring, which may lead to varying patient outcomes, underscoring the importance of standardized practice guidelines [42]. The PELS-1 study published in the *Journal of the American Heart Association* in the same year showed that the in-hospital mortality rate was as high as 60.5% among patients with post-surgical circulatory failure supported by ECMO. However, for patients who survived to discharge, the 1-year, 2-year, 5-year, and 10-year survival rates were 89.5%, 85.4%, 76.4%, and 65.9%, respectively [43]. The study also indicated that patients with complications such as cardiac arrest and intestinal ischemia had significantly lower survival rates, emphasizing the importance of individualized ECMO management [43]. A 2023 study published in *The Journal of Thoracic and Cardiovascular Surgery* found that the short-term survival rate was higher for patients who had ECMO initiated intraoperatively compared with those who had it initiated postoperatively, with in-hospital mortality rates of 57.5% and 64.5%, respectively [44]. The median ECMO usage time was 104 h for patients who had ECMO initiated intraoperatively compared with 139.7 h for those initiated postoperatively, suggesting that selecting ECMO support during surgery may improve postoperative recovery in high-risk patients [44]. Additionally, a 2023 study published in *Annals of Thoracic Surgery* explored the mortality rates and related factors during and after ECMO withdrawal. The study noted that 36.6% of patients died during ECMO support, primarily due to multiple organ failure (38.4%), and 23.1% died after withdrawal, mainly due to persistent heart failure (35.2%), highlighting the importance of timing in ECMO withdrawal [45]. A 2024 study by Chiarini G. et al. found that subclavian/axillary artery cannulation strategies were associated with a higher risk of neurological complications including stroke, intracerebral hemorrhage, and cerebral edema [46]. The complication rate was 19.6% in patients with subclavian/axillary artery cannulation compared with 11.9% in those with femoral artery cannulation (*p* < 0.001), and the risk of seizures was also higher in the subclavian/axillary group (*p* = 0.036), emphasizing the need for postoperative neurological monitoring in these patients [46].

The application of ECMO technology in cardiac surgery-related circulatory failure is crucial; however, it faces challenges. Standardized guidelines, intraoperative ECMO initiation, individualized management, and postoperative neurological monitoring are vital to improving outcomes. In particular, individualized management has become increasingly essential due to the considerable variability in postoperative hemodynamic profiles, anticoagulation strategies, and recovery trajectories observed across ECMO centers. Additionally, the timing of ECMO withdrawal is crucial for reducing complications and mortality. Optimization in these areas is essential to enhance the safety and effectiveness of ECMO in the future.

#### 4.2.4. Prognostic Factors and Patient Selection

Prognostic factors and patient selection have been central issues in ECMO support for circulatory failure research.

Various studies have identified multiple factors closely related to the prognosis of patients receiving ECMO treatment for circulatory failure. First, early hyperoxemia significantly affects the 28-day mortality rate of patients with cardiogenic shock supported by VA-ECMO. In a study involving 430 patients, the 28-day mortality rate was 43%, and hyperoxemia was identified as an independent predictor [47]. Second, ECMO flow management is crucial for preventing complications and improving the short-term outcomes in patients with cardiogenic shock. In one study, 209 patients were categorized into “low flow” and “high flow” groups based on ECMO flow within the first 48 h. The high flow group had higher demands for mechanical ventilation and a higher incidence of ventilator-associated pneumonia (VAP). In contrast, the “low flow” group had a significantly shorter duration of mechanical ventilation compared with the high flow group (4 days vs. 6 days, *p* = 0.009) [48]. Third, the etiology of shock is another important factor. Studies have shown that the five-year survival rate of patients with cardiogenic shock is significantly lower than that of patients with other types of shock, suggesting the need for personalized treatment strategies based on the type of shock [49]. Finally, sepsis, advanced age, and acid–base imbalance are key factors influencing in-hospital mortality in patients with fulminant myocarditis [50]. Data indicate that sepsis increases the risk of death by 2.4 times in patients with fulminant myocarditis treated with ECMO, highlighting the importance of early identification and intervention of these high-risk factors [50]. It is evident that the prognosis of patients receiving ECMO support for circulatory failure is influenced by multiple factors including the etiology of shock, individual characteristics, technical choices, and management strategies.

Given the high cost of ECMO treatment, frequent complications, and complex factors affecting patient prognosis, accurate patient selection is essential. Researchers have begun attempting to construct prognostic prediction models to meet this need. In a systematic review by Giordano et al. [51], 26 ECMO prediction models were compiled, among which the SAVE score” and the “evaluation of novel cardiac and acute surgery to predict mortality and morbidity (ENCOURAGE) score” were two of the most influential prognostic prediction models for ECMO-supported circulatory failure. The SAVE score is based on data from the global ELSO database and was specifically developed for patients with cardiogenic shock supported by VA-ECMO [17]. This score incorporates multiple variables, such as age, disease state, and hemodynamic support needs, effectively distinguishing between high-risk and low-risk patients and optimizing resource allocation and patient selection [17]. The ENCOURAGE score focuses on patients with cardiogenic shock caused by acute myocardial infarction and predicts intensive care unit mortality through seven independent prognostic factors such as age, gender, and lactate levels [18].

However, traditional models have limitations in predictive capability, strong data dependency, and insufficient applicability, making them challenging to use widely in clinical practice. In contrast, machine learning methods handle complex nonlinear relationships, automatically select and extract relevant features, offer high predictive accuracy, broad adaptability, and provide real-time, personalized survival predictions. Therefore, researchers have begun using machine learning techniques to develop new predictive tools such as the ECMO PAL model and the conditional inference trees (CITs) model. The ECMO PAL model, developed by a team at Monash University, utilizes deep neural networks to improve the survival prediction accuracy for VA-ECMO patients by analyzing global big data [52]. The CITs model employs recursive partitioning and statistical analysis methods, making it suitable for multicenter clinical data and providing a more interpretable and robust survival prediction tool [53]. These new methods, with their more precise personalized predictions, overcome the limitations of traditional models and advance precision medicine in ECMO treatment. Importantly, these innovations not only enhance prognostic accuracy, but also support the implementation of individualized ECMO management strategies tailored to patient-specific risks, conditions, and therapeutic responses.

#### 4.2.5. Application of ECMO in Ventricular Tachycardia Ablation

In addition to the above scenarios, ECMO has also been increasingly adopted to support patients undergoing high-risk electrophysiological interventions, particularly catheter ablation of hemodynamically unstable ventricular tachycardia (VT) or electrical storm. A contemporary overview by Mariani et al. summarized the clinical role of mechanical circulatory support, including VA-ECMO, in maintaining circulatory stability during VT ablation in patients with severely impaired cardiac function [54]. By enabling continuous organ perfusion and improving procedural tolerance, ECMO facilitates safe substrate mapping and minimizes periprocedural complications. Although ECMO does not directly reduce arrhythmia recurrence, it significantly enhances procedural safety and short-term outcomes in this highly vulnerable population. This expanding application underscores ECMO’s evolving role as not only a rescue modality, but also a tailored peri-procedural adjunct in complex electrophysiological care.

#### 4.2.6. Underrepresentation of Complications in Keyword Analysis

Despite the critical impact of complications, such as bleeding, thrombosis, and limb ischemia, on ECMO outcomes, our keyword analysis did not identify these terms among the most frequently occurring or central keywords. This apparent absence may result from the substantial terminological heterogeneity used across studies. For example, “bleeding”, “hemorrhage”, and “coagulopathy” may refer to overlapping concepts but appear as separate terms, diluting their co-occurrence strength. Similarly, “limb ischemia” may be described as “lower extremity hypoperfusion” or “vascular access complication”, leading to fragmentation in keyword frequency. Nonetheless, complications remain a major focus in recent ECMO-related research. Ahmad et al. demonstrated that mechanical unloading with IABP or Impella during VA-ECMO significantly increased the risk of bleeding, particularly with Impella support [55]. Meanwhile, Shen et al. proposed a modified remote infusion catheter technique that effectively reduced the incidence of acute limb ischemia in peripheral VA-ECMO cases [56]. These findings emphasize the importance of addressing complication-related themes explicitly in future bibliometric models, potentially through the construction of targeted sub-networks to capture their evolving significance.

## 5. Limitations of the Study

Missing research hotspot on complications: Complications associated with ECMO treatment are a crucial research direction in this field. However, in the analysis of the highly cited literature bursts, only one paper on complications (Cheng R., 2014 [57]) appeared, which was published in 2014. Complication-related research did not stand out among the hot keywords in the past five years, possibly due to the diverse nature of complications, leading to the low frequency of related keywords and failure to form a significant research trend in the analysis.

Limitations in study content: This study did not include biomarker-related or disease-specific keywords (e.g., lactate, troponin, fulminant myocarditis, acute myocardial infarction) in the keyword co-occurrence or citation burst analysis. These variables are clinically significant because biomarkers are essential for early risk stratification, patient selection, and outcome prediction during ECMO support. Similarly, different disease etiologies may influence ECMO indications, management strategies, and prognosis. The omission of such factors may result in an incomplete representation of the research landscape. Future bibliometric studies should consider integrating these dimensions to provide a more nuanced and clinically relevant analysis.

Time limitations: Although this study’s data covered a long-time span, the impact of emerging research may not be reflected in a timely manner, particularly innovative research directions that have emerged in recent years. As the popularity of these studies is still growing, they may not have received adequate attention in this analysis.

Data source limitations: This study was based solely on the Web of Science Core Collection, which may not comprehensively cover all of the relevant literature, especially studies published in other languages or regions. For example, the non-English literature or significant studies published in certain regions may have been overlooked, potentially limiting the analysis results.

## 6. Conclusions

This study employed bibliometric methods to analyze the academic influence, research trends, and hotspots in the field of ECMO support for circulatory failure from 1945 to 2024. Our analysis shows that since the 1990s, research related to ECMO has grown significantly, with the United States maintaining a dominant position in this field, while the scientific influence of China, South Korea, and Saudi Arabia has rapidly risen in recent years. China should continue to strengthen their international collaboration and improve their research quality to enhance its global influence, academic standing, and international voice in this field. Recently, areas such as cardiac arrest, post-cardiac surgery circulatory failure, left ventricular unloading, and prognostic factors have become hotspots in ECMO research. Research in these areas reflects key challenges in current clinical practice and offers insights for future technological optimization and improvements in treatment strategies.

## Figures and Tables

**Table 1:** Annual frequency of top 20 keywords in ECMO support for circulatory failure (2020–2024).

Key Word	2020	2021	2022	2023	2024	Total
Extracorporeal membrane oxygenation	229	265	267	259	182	1202
Cardiogenic shock	76	99	121	84	76	456
Cardiopulmonary bypass	78	68	83	68	27	324
Congenital heart disease	63	69	65	67	31	295
Cardiac surgery	57	58	68	55	35	273
Mechanical circulatory support	34	57	63	51	42	247
Cardiac arrest	49	51	53	60	34	247
VA-ECMO	32	38	61	45	47	223
Outcomes	44	44	40	32	26	186
Heart transplantation	28	44	40	39	25	176
Mortality	25	37	43	39	16	160
Heart failure	33	37	35	28	26	159
Pediatrics	31	41	28	35	24	159
Extracorporeal cardiopulmonary resuscitation	25	35	32	32	31	155
Left ventricular assist device	37	30	38	26	16	147
Coronary artery bypass grafting	28	29	25	37	20	139
Cardiopulmonary resuscitation	32	16	23	28	28	127
COVID-19	20	38	34	21	9	122
Shock	18	22	25	32	7	104
Impella	16	22	22	20	18	98

Although biomarkers such as “lactate” and “troponin” as well as disease-specific terms like “fulminant myocarditis” and “acute myocardial infarction” are clinically significant in ECMO management, these terms were underrepresented in the co-occurrence and citation burst analyses. This may be attributed to inconsistent terminology usage, varying reporting practices, and their frequent embedding within broader clinical contexts rather than as standalone topics. Future bibliometric efforts should aim to standardize and cluster such clinically important terms to better capture their academic relevance and thematic evolution.

## Data Availability

The original data presented in the study are openly available in [Web of Science Core Collection (SCIE)] at https://www.webofscience.com (accessed on 8 August 2024).

## List of Abbreviations

CPB	Cardiopulmonary Bypass
CITs	Conditional Inference Trees
CPC	Cerebral Performance Category
ECPR	Extracorporeal Cardiopulmonary Resuscitation
ECMO	Extracorporeal Membrane Oxygenation
ELSO	Extracorporeal Life Support Organization
SCIE	Science Citation Index Expanded
SAVE	Survival After Veno-Arterial ECMO (score)
PAL	Prediction and Analysis of Long-term outcomes
VOS	Visualization of Similarities (used in VOSviewer)
VA-ECMO	Veno-Arterial Extracorporeal Membrane Oxygenation
VAP	Ventilator-Associated Pneumonia
